# A Microfluidic Device for Preparing Next Generation DNA Sequencing Libraries and for Automating Other Laboratory Protocols That Require One or More Column Chromatography Steps

**DOI:** 10.1371/journal.pone.0064084

**Published:** 2013-07-24

**Authors:** Swee Jin Tan, Huan Phan, Benjamin Michael Gerry, Alexandre Kuhn, Lewis Zuocheng Hong, Yao Min Ong, Polly Suk Yean Poon, Marc Alexander Unger, Robert C. Jones, Stephen R. Quake, William F. Burkholder

**Affiliations:** 1 Institute of Molecular and Cell Biology, Agency for Science, Technology and Research, Proteos, Singapore; 2 Fluidigm Corporation, South San Francisco, California, United States of America; 3 Departments of Bioengineering and Applied Physics, Stanford University and Howard Hughes Medical Institute, Stanford, California, United States of America; 4 Visiting Investigator, Institute of Molecular and Cell Biology, Agency for Science, Technology and Research, Proteos, Singapore, Singapore; German Cancer Research Center, Germany

## Abstract

Library preparation for next-generation DNA sequencing (NGS) remains a key bottleneck in the sequencing process which can be relieved through improved automation and miniaturization. We describe a microfluidic device for automating laboratory protocols that require one or more column chromatography steps and demonstrate its utility for preparing Next Generation sequencing libraries for the Illumina and Ion Torrent platforms. Sixteen different libraries can be generated simultaneously with significantly reduced reagent cost and hands-on time compared to manual library preparation. Using an appropriate column matrix and buffers, size selection can be performed on-chip following end-repair, dA tailing, and linker ligation, so that the libraries eluted from the chip are ready for sequencing. The core architecture of the device ensures uniform, reproducible column packing without user supervision and accommodates multiple routine protocol steps in any sequence, such as reagent mixing and incubation; column packing, loading, washing, elution, and regeneration; capture of eluted material for use as a substrate in a later step of the protocol; and removal of one column matrix so that two or more column matrices with different functional properties can be used in the same protocol. The microfluidic device is mounted on a plastic carrier so that reagents and products can be aliquoted and recovered using standard pipettors and liquid handling robots. The carrier-mounted device is operated using a benchtop controller that seals and operates the device with programmable temperature control, eliminating any requirement for the user to manually attach tubing or connectors. In addition to NGS library preparation, the device and controller are suitable for automating other time-consuming and error-prone laboratory protocols requiring column chromatography steps, such as chromatin immunoprecipitation.

## Introduction

The field of microfluidics, or miniaturized plumbing, has as one of its goals the automation of laboratory assays and protocols. This is often termed “lab on a chip”, and substantial progress has been made toward achieving this goal [1]. In the area of molecular biology, early proof-of-principle implementations of microfluidics-based protocols for cell lysis and cDNA preparation have demonstrated the potential for what can be done [2–9]. However, challenges remain, and the full value of microfluidic devices for large-scale automation will not be realized until the ability to flexibly implement molecular biology or biochemistry protocols that involve multiple steps has been demonstrated. This paper describes a novel microfluidic device, the automated multi-column chromatography (AMCC) chip, capable of performing an arbitrary number of serial reaction-purification steps on 16 independent samples. We used this device to implement protocols for generating high quality Next Generation DNA sequencing libraries from bacterial and human genomic DNA samples.

Next generation sequencing (NGS) sample library preparation is a growing and important application where the benefits of microfluidics-based automation could be quite powerful. Advances in sequencing methodologies have brought about a paradigm shift in biomedical sciences [10], with a profound impact on the understanding of genetic variations [11,12], and improved clinical assessments [13]. Several NGS techniques have emerged [14–16], providing platforms for high throughput generation of massive numbers of short reads capable of providing high genome coverage [17,18]. With the advent and affordability of personal benchtop sequencers such as the Ion Torrent Personal Genome Machine (PGM) and Illumina MiSeq, these machines are becoming routine lab tools. As the cost of sequencing has decreased exponentially, for many experiments the cost of library preparation now equals the cost of sequencing. As a result, an enormous amount of manual effort is spent on the molecular biology steps required to create sequencing libraries and more often than not, several libraries need to be generated in parallel at any one time. For instance, the PGM is capable of handling three sequencing runs consecutively on the 314 chip per workday without a change in reagents.

The flexibility of the AMCC is shown by the fact that standard library preparation protocols for both the Ion Torrent PGM and Illumina MiSeq were directly implemented on chip, and the products of these preps led to high quality sequencing results on both NGS platforms. The AMCC chip utilizes integrated micro-valves [19,20] to ensure precise liquid handling on the device. Every reaction therefore requires only nanoliter volumes which presents significant savings on reagent usage. Samples and reagents are directly pipetted onto a custom carrier. An accompanying benchtop instrument automates the loading and mixing of different reagents as required for the library preparation protocol. One additional benefit of this approach is to eliminate the need to premix reagents before loading on chip, providing greater ease during library generation and reducing opportunities for operator error. Using the device, multiple sequencing libraries were produced reliably with minimal handling time compared with the conventional hands-on approach.

## Materials and Methods

### Device design and operation

**Figure 1:**
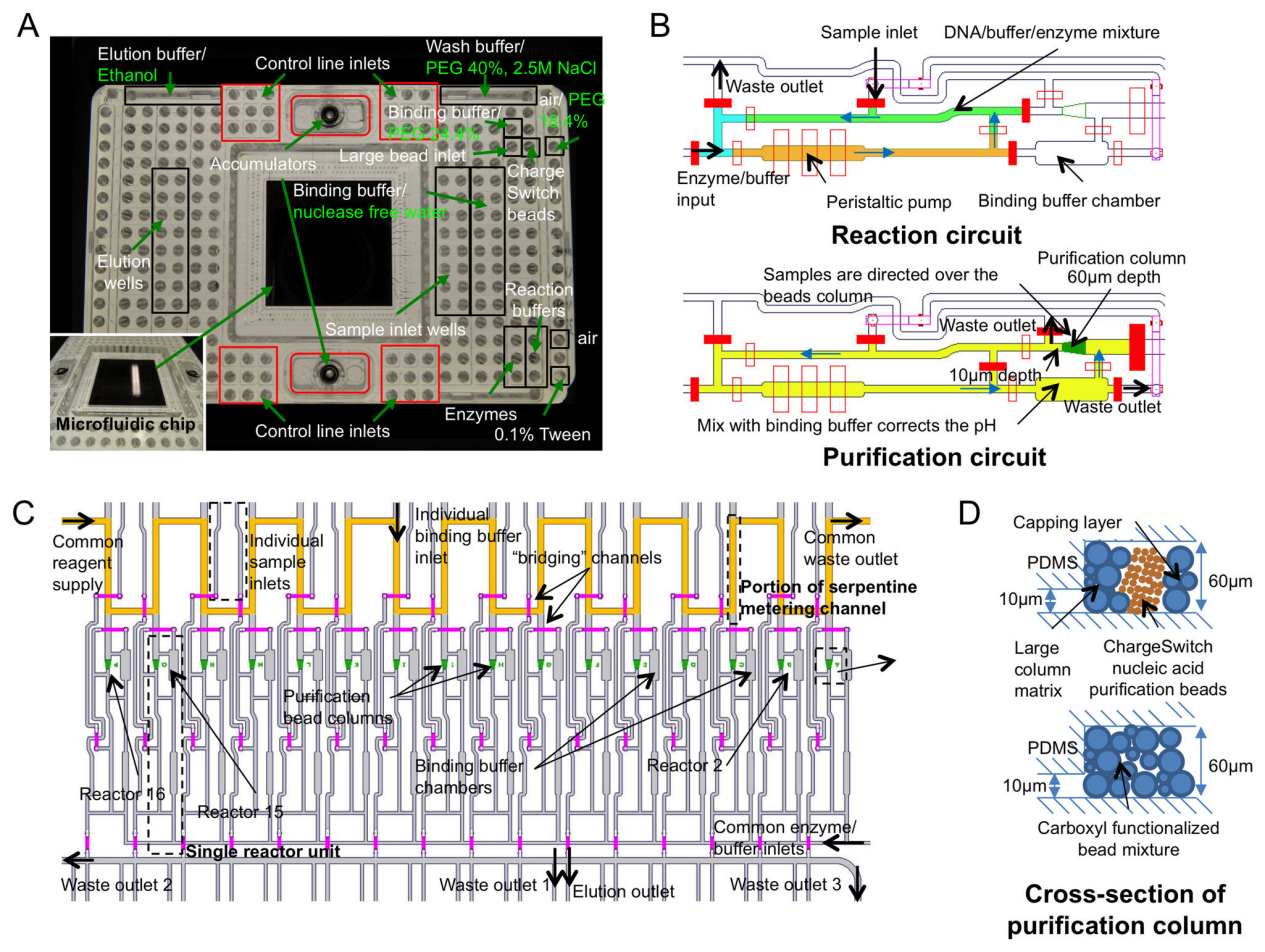
Microfluidic device for automated NGS library preparation. (a) Automated multi-column device mounted on a plastic carrier that provides wells for loading samples and reagents and for pressurized operation of the device. The wells used to load reagents for NGS library preparation are labeled. Chromatography columns for the selective binding and release of DNA were formed either with ChargeSwitch beads or with carboxylated beads. Reagents that were used exclusively with the carboxylated beads are labeled in green. (b) Schematic of single reactor unit for reaction mixing and DNA purification. The regions denoted in the reaction circuit are as follows: Green, Sample; Orange, Buffer; Blue, Enzyme. Red solid rectangular boxes represent activated valves that partition the individual circuits. (c) Parallelization of 16 reactors on chip for preparation of up to 16 independent libraries. Layout of the entire device without the valve map showing reagent inlets and the design for multiplex library generation. The serpentine metering channel designed to ensure reliable column packing is highlighted in orange. (d) Schematics showing cross-sections of purification columns loaded with either 1) ChargeSwitch beads, which are held in place with a frit layer and a cap layer formed by larger beads, or 2) carboxylated beads.

The AMCC chip was designed to enable the automation of multistep laboratory protocols that combine one or more column chromatography steps with steps in which reagents are mixed and incubated. The chip is a prototype integrated fluidic circuit (IFC) developed and provided by Fluidigm Corp, South San Francisco. It was made using multi-layer soft lithography (MSL) methods [19] and contains independent modules for processing and recovering 16 different samples in parallel. For ease of use, the chip is mounted on a plastic carrier that contains wells for loading samples and reagents and for recovering processed samples (Figure 1A). The size of the carrier and the spacing between wells are the same as a standard 384 well plate, conforming to the Society for Biomolecular Screening (SBS) microplate format [23] and is thus compatible with standard multichannel pipettors and robotic workstations. In the current design, the chip provides two wells for the bead slurry used to form the column matrix, six wells for reagents used in the protocol (labeled "Enzymes" and "Reaction buffers" in Figure 1A), and wells for the binding, wash, and elution buffers used in the column chromatography steps, including four large-volume wells of 175 µl each (Figure 1A). In addition, 36 wells connect to the control lines used to actuate the microfluidic valves on the device, and two wells are used to purge channels with air.

After the samples and reagents have been aliquoted into the appropriate wells of the carrier, the device is placed in a programmable robotic workstation - the IFC controller, also provided by Fluidigm Corp. - which operates the device by pressurizing the well inlets to inject reagents and actuate valves [24]. The IFC controller also provides programmable temperature control for the whole chip over the range 4-98 ^°^C, and the carrier-mounted chip is backed with a silicon integrated heat spreader for uniform and efficient heat transfer. Once a protocol has been completed, an accumulator valve on the carrier is closed to ensure that the microfluidic valves separating the sample elution wells from the rest of the chip remain closed (Figure 1A). The device is then removed from the controller, and the processed samples are recovered from the elution wells by pipetting.

To enable multiple enzymatic and column purification steps to be performed on a sample, each sample module contains two interconnected fluidic loops, termed the reaction circuit and the purification circuit (Figure 1B). The reaction circuit includes an inlet for introducing the sample and an inlet for introducing reagents from the wells labelled "Enzymes" and "Reaction buffers" (Figure 1B, 1C, and S1 in File S1). The purification circuit includes two chambers, one for packing the column matrix and the other for mixing the sample with binding buffer before loading onto the column (Figure 1B, 1C, S2A in File S1), An inlet feeding into the top of the column chamber is used to load beads and to introduce buffers onto the column. By reversing the flow and setting appropriate valves, the column inlet can also be used as an outlet, allowing for procedures such as washing out beads packed in the column so that they can be replaced with another type of bead for a subsequent column chromatography step. A peristaltic pump formed by three valves is used to mix reagents in the reaction circuit and to pump reagents over the column in the purification circuit. Three outlet valves placed at different positions around the reaction and purification circuits can be used to remove fluid, directing it either to waste or to the sample elution well. Fluid flow is directed through the reaction circuit or through the purification circuit by setting appropriate valves.

The reaction circuit can be partitioned into two or three separate chambers by actuating valves placed at different points around the circuit. The three isolated chambers are referred to as the "sample", "enzyme", and "buffer" chambers (Figure S1 in File S1). The sample chamber can be filled either from the sample inlet or with sample eluted from the column. In parallel, the enzyme and buffer chambers can be loaded with reagents or washed and purged with air before being loaded with new reagents for a subsequent step of the protocol. When ready, the valves separating the chambers within the reaction circuit are opened, and the reagents are mixed by activating the peristaltic pump and circulating the reagents around the reaction circuit. In order to capture the sample on the column, the sample is first mixed with binding buffer and then directed into the purification circuit and over the column (Figure 1B, S2A in File S1). A continuous flow can be directed from the sample inlet over the column, through the outlet below the column, and then to the waste collection well on the carrier in order to load and concentrate dilute samples on the column. Alternatively, a sample in binding buffer can be circulated through the purification circuit and over the column multiple times to try to maximize the amount of sample bound.

### Design of the column chamber and column packing

Prior methods of column generation on microfluidic devices require extensive preparation of the bead slurry [25–27] or optical feedback to monitor the column packing process [20]. This increases the hands-on time and skill required to accurately maintain consistency between columns. In some devices described previously, column construction was done serially, which takes longer and limits throughput [3,20]. Promising alternative methods of forming bead columns in MSL structures have been demonstrated using partially closed valves - ‘leaky valves’ - to trap beads [3,20] or using bypass channels alongside the main chromatographic column to promote the generation of uniform columns [28,29]. Recent approaches for performing column chromatography on a microfluidic scale have been reviewed extensively [30–33]. Most microfluidic-scale column chromatography devices were designed to automated specific protocols, whereas the AMCC chip was designed to allow for flexible implementation of a variety of different protocols.

Our design employs a serpentine metering channel (Figure 1C) to queue a fixed volume of bead solution (~20 nl) ahead of each empty column, using a valve configuration to isolate and then load all columns in parallel (illustrated in Figure S3A in File S1). The bed volume of the column matrix is controlled by the number of bead packing cycles and the input concentration of the beads. Beads are captured in the column using a simple step down in the channel height from 60 µm to 10 µm, forming a lip (or weir structure) that captures beads larger than 10 µm (Figure 1D). When an application requires that smaller bead sizes be used (for instance, if functionalized beads designed for a specific application are only available in a smaller size) then these beads can be sandwiched between two layers of larger beads that form a 'dynamic frit' and a capping layer that helps to stabilize the column (Figure 1D). The primary factor in column design was to have sufficient packed bead volume to capture a sufficient quantity of DNA. We chose a tapering shape both to assist the dynamic fritting process and to make the loading of the beads more even. We did not systematically optimize the column geometry; once the column packing (dynamic fritting and capping) was established, column performance was robust and sufficient for the reactions.

Two types of bead columns were investigated for their suitability in NGS library preparation: ChargeSwitch beads (Invitrogen) and carboxylated microspheres. ChargeSwitch beads are positively charged at low pH (< 6.5) and bind DNA. At higher pH (> 8.5), the beads are neutral and DNA is released. Washes are performed at an intermediate pH (~7). Carboxylated beads preferentially bind DNA onto their surface in the presence of high concentrations of PEG and NaCl. DNA is released when the concentrations of PEG and NaCl are reduced or when the beads are washed with water. Since ChargeSwitch beads are much smaller than the outlet from the column chamber (~0.5-1 µm), we prepared columns on-chip by first loading a mixture of 15, 6, 4 and 2 µm beads (Life Technologies, USA) to form a frit, then loaded the ChargeSwitch beads, and finally loaded a capping layer of the large bead mixture (Figure 1D. The frit layer of large polystyrene beads was created using six cycles of metering the serpentine, followed by six cycles of ChargeSwitch beads, and two cycles of large polystyrene beads to form the capping layer. In the case of carboxylated beads, only one type of slurry comprising 15, 6, 4 and 2 µm beads is created using six cycles of serpentine metering. In both case, the column formation was preceded by a coating of the channels with Tween 20 0.1% [Promega, USA] in order to reduce beads adhesion to PDMS channels walls.

### DNA samples and shearing of genomic DNA

Genomic DNA from *E. coli* strain DH10B was obtained from the Ion Control Materials Kit (Life Technologies, USA), and genomic DNA from the human U-2 OS osteosarcoma cell line (ATCC HTB-96) [21] was a kind gift from Dr. Ernesto Guccione (Institute of Molecular and Cellular Biology, A*STAR). Genomic DNA was fragmented prior to loading on the AMCC chip. For Illumina library preparation, 2 µg of genomic DNA was fragmented by physical shearing using the Covaris instrument (Covaris Inc., USA) at a duty cycle of 10%, intensity of 5, and 200 cycles per burst for 430 s. For Ion Torrent library preparation, enzymatic shearing was performed using the IonShear Reagents kit (Life Technologies, USA) according to the manufacturer’s recommendation: 2 tubes, each containing 1 µg genomic DNA, reaction buffer, and IonShear enzyme mix, were incubated at 37 ^°^C for 15 min. Stop buffer was added to terminate the reactions, and fragmented DNA was purified using the Qiagen MinElute PCR purification kit (Qiagen, USA). The Covaris and IonShear protocols typically yielded similar size distributions of fragmented DNA ranging from 50–500 bp with a peak around 150 bp.

### Reagents and device loading

Reagents and samples required for library preparation were pipetted onto a custom carrier attached to the microfluidic device (Fig. 1A). Care was taken throughout the loading process to avoid introducing air bubbles into the wells, as that would affect operation of the device. Fragmented genomic DNA (100 ng/µl) was mixed 1:1 (v/v) with binding buffer. The binding buffer used with ChargeSwitch bead columns was 50 mM citrate buffer-NaOH, pH 5.0 [Teknova, USA], 1% Tween 20 [Promega, USA] and 1 mM EDTA [Teknova, USA], and the binding buffer used with carboxylated bead columns was 40% PEG, 2.5M NaCl. DNA samples (1 µL) were loaded into 14 of the 16 available sample inlet wells (larger volumes can also be used but would take longer to load onto the chip). An equal volume of binding buffer was loaded into the two remaining sample inlet wells as "no DNA" controls to assess if any leakage or spill-over between sample modules occurred. Next, 5 µl of each enzyme mix and 25 µl of each reaction buffer were loaded into the designated wells. End repair, dA tailing, and adapter ligation were performed using the NEBNext End Repair Module (10,000 units/ml T4 polynucleotide kinase; 3,000 units/ml T4 DNA polymerase), the NEBNext dA-tailing module (Klenow Fragment [3’ → 5’ exo^-^], specific activity not stated), and the NEBNext Quick Ligation module (Quick T4 DNA Ligase, specific activity not stated; New England Biolabs, USA). The reaction buffers supplied with the End repair kit (50 mM Tris-HCl, pH 7.5; 10 mM MgCl_2_; 10 mM dithiothreitol [DTT]; 1 mM ATP; 0.4 mM dATP; 0.4 mM dCTP; 0.4 mM dGTP; 0.4 mM dTTP, final concentrations) and the dA tailing kit (10 mM Tris-HCl, pH 7.9; 10 mM MgCl_2_; 50 mM NaCl; 1 mM DTT; 0.2 mM dATP, final concentrations) were diluted to 1x concentration and, at the same time, supplemented with bovine serum albumin (1 mg/ml final concentration) and Tween 20 (0.5% v/v final concentration; Promega, USA) to minimize loss of the enzymes by adsorption to the polymeric micro-channel walls. The reaction buffer supplied with T4 DNA ligase (66 mM Tris-HCl, pH 7.6; 10 mM MgCl_2_; 1 mM DTT; 1 mM ATP, 6% polyethylene glycol 6000) was supplemented with pre-annealed dsDNA sequencing adapters (50 nM final concentration) specific for the Ion Torrent PGM (modified adapters A [5’-Phos/CTGAGTCGGAGACACGCAGGGATGAGATGG*C-3’, 5’-CCATCTCATC CCTGCGTGTCTCCGACTCAG*T-3’] and P1 [5’-CCACTACGCCTCCGCTTTCCTCTCTATGGGCAGTCGGTGAT*T-3’; 5’-Phos/ATCACCGACTGCCCATAGAGAGGAAAGCGGAGGCGTAGTGG*C-3’]) or Illumina platform (PE adapters [5'-Phos/GATCGGAAGAGCGGTTCAGCAGGAATGCCGAG-3'[and [5'-ACACTCTTTCCCTACACGACGCTCTTCCGATCT-3']). The Ion Torrent and Illumina adapters were annealed in a thermocycler using the following parameters: 97°C for 2 min followed by a 1°C reduction every minute until 25°C; 25^°^C for 5 min; hold at 4˚C.At this stage, the carrier was centrifuged briefly to ensure that the small volumes of the samples and enzyme mixes entered the chip.

Water was added to the control line inlets and the buffers for binding, washing, and eluting sample DNA were loaded into the wells indicated in Figure 1A. The buffers used with ChargeSwitch bead columns were: binding buffer (50 mM citrate buffer-NaOH, pH 5.0 [Teknova, USA], 1% Tween 20 and 1 mM EDTA [Teknova, USA]); wash buffer (distilled water, supplied with the ChargeSwitch bead kit; Life Technologies, USA); and elution buffer (20 mM Tris-HCl, pH 9.0; Teknova, USA). The buffers used with carboxylated microsphere columns were: PEG 24.4%; PEG 40% + 2.5M NaCl; PEG 18.4%; nuclease free water; and ethanol. The small wells were loaded with 30 µl of each solution and the large wells were loaded with 150-200 µl of wash buffer or elution buffer. The beads used to form the column matrix were loaded last to prevent sedimentation before the start of library preparation. 30 µl of each bead slurry was added to the designated wells.

To prepare columns using ChargeSwitch beads, the mixture of large and small beads consisted of 2, 4, 6 and 15 µm polystyrene beads (Flow cytometry size calibration kit, Life Technologies, USA). The individual bead slurries provided by the manufacturer were mixed in the ratio of 4:4:4:1 (v/v) and were diluted 1:5 (v/v) in 10 mM Tris-HCl, pH 9, 10% PEG 8000, 0.1 mM EDTA, and 0.05% Tween 20. The slurry of ChargeSwitch beads provided by the manufacturer (25 mg/ml in 10 mM MES, pH 5.0,10 mM NaCl, 0.1% Tween 20) was mixed with binding buffer in the ratio 1:10 (v/v). The final ratio of bed volume to total volume in the slurry of ChargeSwitch beads loaded onto the chip was approximately 1:110 (v/v).

To prepare columns using carboxylated microspheres, the bead slurry was prepared by mixing microspheres of 6 µm (Polysciences Inc., USA), 4.5 µm (Polysciences Inc., USA) and 3 µm (Polysciences Inc., USA) with 15 µm polystyrene beads (Life Technologies, USA) in the ratio 4:4:4:1 (v/v) and diluted in same buffer used earlier.

Immediately after aliquoting the bead slurries, the carrier-mounted device was loaded onto a robotic workstation for programmable operation of the device. The device was operated at room temperature. Completed libraries were eluted into the collection wells in a volume of 1 µl for experiments using ChargeSwitch bead columns and 13 µl for carboxylated bead columns. These were recovered by pipetting off chip.

### Performing DNA purification and size selection on the AMCC chip using carboxylated beads

Size selection is a tedious and time-consuming step that is generally required after NGS library preparation to remove DNA fragments in the library that are too short or too long and would otherwise interfere with sequencing. We therefore sought to incorporate size selection into the NGS library preparation workflow implemented on the AMCC chip. We took advantage of recent reports that size selection can be performed using carboxylated beads to bind DNA and titrating the concentration of polyethylene glycol (PEG) to selectively elute DNA fragments of different sizes [34–36]. In pilot titration experiments, we determined that we could recover DNA fragments in the size range 150-500 bp by using 12.2% PEG to adsorb DNA fragments larger than 150 bp to carboxylated beads, washing away smaller DNA fragments, and then using, 9.2% PEG to elute DNA fragments smaller than 500 bp for collection.

### Manual library size selection

Manual size selection of the sequencing libraries was performed using 2% agarose gels. The range of fragment sizes selected for sequencing depended on the platform: 180-210 bp (Ion Torrent) and 200-500 bp (Illumina). Gel extraction was performed using the Qiagen MinElute gel extraction kit (Qiagen, USA).

### Quantification, amplification, and sequencing

The concentration of size-selected sequencing libraries was determined by real-time quantitative PCR (RT-qPCR) on a Stratagene MX3005p using the Maxima SYBR Green qPCR master mix (Fermentas Inc., USA) or the Illumina library quantification kit (Kapa Biosystems, USA). When size-selected libraries were quantified immediately after size selection and before they had been amplified by PCR, 10% of the library was used as a template in the RT-qPCR reactions using Maxima SYBR Green qPCR master mix. 0.3 µM of each adapter specific primer (Ion torrent forward: 5’-CCATCTCATCCCTGCGTGTCTCCGACTCAG-3’, Ion torrent reverse: 5’-CCGCTTTCCTCTCTATGGGCAGTCGGTGAT-3’; MiSeq forward: 5’-ACACTCTTTCCCTACACGACG-3’, MiSeq reverse: 5’-CTCGGCATTCCTGCTGAAC-3’) were added to samples generated for the Ion Torrent or Miseq platform. RT-qPCR was performed with the following thermal cycling conditions: 1 cycle of 95°C for 30 s and 35 cycles consisting of 95°C for 10 s, 60°C for 30 s and 72°C for 30 s. Fluorescence signals for two channels using SYBR Green (495 nm) and ROX (535 nm) were recovered. DNA concentrations were calculated from the RT-qPCR data based on a standard curve generated from serially diluted genomic DNA of known concentration. The DNA concentrations of the standard curve samples were determined using the NanoDrop 8000 spectrometer (ThermoScientific, DE, USA). For the quantification of MiSeq libraries after amplification, this was performed as per manufacturer’s protocol using PE PCR Primer 1.0 (5’-AATGATACGGCGACCACCGAGATCTACACTCTTTCCCTACACGACGCTCTTCCGATCT-3’) and PE PCR Primer 2.0 (5’-CAAGCAGAAGACGGCATACGAGATCGGTCTCGGCATTCCTGCTGAACCGCTCTTCCGATCT-3’).

Ion Torrent sequencing was performed on the Ion Torrent Personal Genome Machine (PGM; Life Technologies, USA) according to the manufacturer’s protocols. Size-selected sequencing libraries were amplified by PCR using a proprietary primer mix provided with the IonXpress fragment kit (Life Technologies, USA), and the DNA concentrations of the amplified libraries were determined using a high sensitivity DNA chip (Agilent High Sensitivity DNA Kit) on the Agilent 2100 Bioanalyzer (Agilent Technologies, USA). Samples were diluted to an estimated concentration of 70 million molecules in 5 µl of nuclease free water, and emulsion PCR reactions were performed on the Ion Torrent One Touch system (Life Technologies Corp., USA) following the 100 bp template preparation protocol (Rev. E; revision date: 27th Oct 2011). Sequencing was performed using Ion 314 chips (Life Technologies, USA) with an estimated throughput of 10 Mbp or higher. Raw fastq files generated from the base calls by the Ion Torrent software suite (version 2.0.1) were used for genome alignment.

Illumina sequencing was performed on the Illumina MiSeq (Illumina, USA) according to the manufacturer’s protocol. Size-selected libraries were amplified by PCR for 18 cycles as per manufacturer’s protocol and denatured DNA was diluted to 8 pM final concentration for loading onto the flow cell. Libraries were sequenced using paired-end 25 bp reads. Raw fastq files generated by the Real Time Analysis software (version 1.13.56.0) on the MiSeq were used for genome alignment.

### Preparing sequencing libraries from *E. coli* genomic DNA

To assess the performance and reliability of the AMCC chip for preparing NGS sequencing libraries from bacterial genomic DNA, we performed two runs to prepare libraries for the Ion Torrent PGM platform and one run to prepare libraries for the Illumina MiSeq platform with the ChargeSwitch bead column configuration. For each run, we loaded 100 ng of fragmented genomic DNA from *E. coli* strain DH10B into 14 of the 16 sample wells. (Although we loaded aliquots of the same DNA sample in the 14 reactors for the proof-of-principle experiments described here, a more typical application would be to load and process up to 16 different samples on the same chip.) We filled the remaining two sample wells on each device with buffer, to determine if there was any leakage or spillover of samples between modules.

Except for the use of platform-specific adapters, the reagents and on-chip protocols were essentially the same for the AMCC chip runs to prepare Ion Torrent and Illumina libraries. We also prepared sequencing libraries for the Ion Torrent and Illumina platforms manually using the standard benchtop protocols recommended by the manufacturers to compare the quality of the sequencing data obtained from libraries prepared manually with those prepared using the AMCC chip.

After the completion of the on-chip and manual library preparation protocols, size selection was performed to obtain the range of fragment sizes recommended for each sequencing platform: 180-210 bp for the Ion Torrent and 200-500 bp for the Illumina MiSeq. Following size selection, we determined the yield of library DNA by RT-qPCR and found that it was relatively uniform across all the sample modules on the AMCC chip that had been loaded with genomic DNA (). Coefficients of variation were 0.49 and 0.32 for MiSeq (A) and Ion Torrent libraries (B) respectively. No DNA was detected in the two sample modules on each AMCC chip that served as buffer-only controls, indicating that there was no detectable spillover or leakage of samples between modules. The amount of DNA recovered for the MiSeq libraries (average of 1.53 ng) was significantly higher than for the Ion Torrent libraries (average of 0.38 ng), which is due in part to our having purified a larger range of fragment sizes during size selection. The low yield of MiSeq library DNA from sample module 8 (A) can be attributed to an air bubble in the sample inlet, which subsequently entered the reactor. This was an isolated incident and can be avoided if care is taken while pipetting the samples and other reagents into the wells of the plastic carrier. Following size selection, we amplified the sequencing libraries by PCR (7 cycles for the Ion Torrent libraries and 18 cycles for the Illumina libraries, as recommended by the manufacturers), and we again quantified the yield of library DNA by RT-qPCR (Figure S4 in File S1). The yields for all the libraries prepared on the AMCC chip were more than sufficient for repeated sequencing runs.

**Figure 2:**
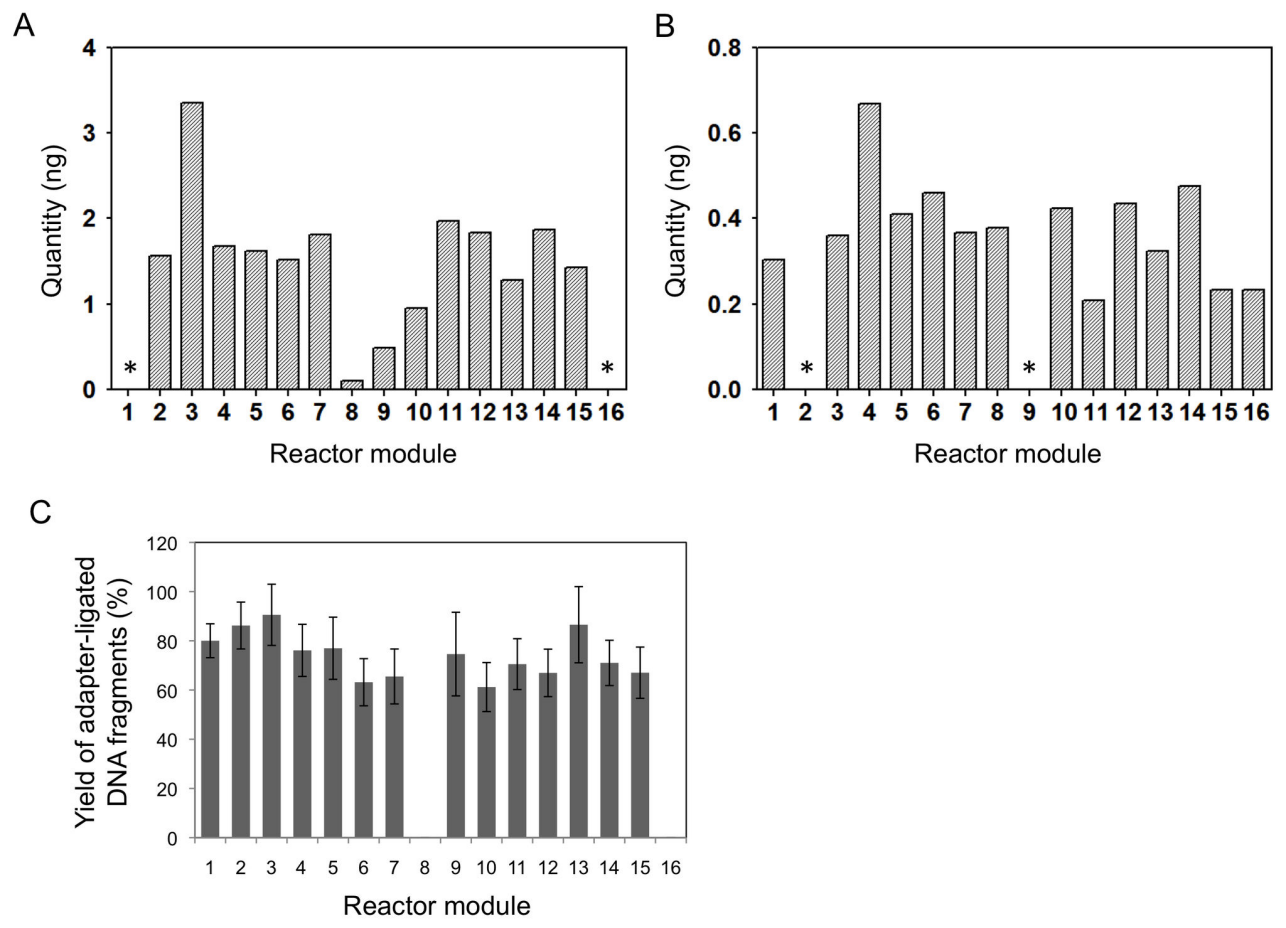
Quantification of *E. coli* strain DH10B library DNA after size selection. (a) Illumina libraries; (b) Ion Torrent libraries. Asterisks indicate sample modules where buffer was loaded instead of genomic DNA. (c) Efficiency of library preparation reactions on the AMCC chip. The percentage of *E. coli* DNA fragments with Illumina sequencing adapters ligated onto both ends was estimated by RT-qPCR. The amount of *E. coli* genomic DNA present was determined by RT-qPCR with primer pairs recognizing six regions of the *E. coli* genome, and the amount of library DNA with adapters ligated onto both ends was determined by RT-qPCR with a primer pair recognizing the Illumina sequencing adapters. RT-qPCR data were converted to nanograms of DNA using standard curves (Materials and Methods). The estimated amounts of *E. coli* genomic DNA present in each library varied somewhat between the six locus-specific RT-qPCR reactions, so the bar graph indicates the mean values, and the error bars indicate the standard error of the mean.

### Preparing sequencing libraries from human genomic DNA

We set about evaluating the performance of the AMCC chip with human genomic DNA, a more complex genomic sample consisting of more than 3 billion nucleotide bases. Illumina MiSeq sequencing was selected as its throughput on the 2 by 150 bp kit was higher than that achieved on the Ion Torrent PGM running a 318 chip [18]. Fragmented genomic DNA from U-2 OS osteosarcoma cells (100 ng) was loaded into 14 of the 16 wells on the chip without prior size selection. Illumina MiSeq pair-end adapters were ligated and subsequently size-selected for 200-500 bp.

### Manual library preparation

For comparison, sequencing libraries were prepared off-chip (manually) using the standard protocols recommended by Ion Torrent (using the IonXpress fragment library kit, Life Technologies, USA) and Illumina (using reagents from New England Biolabs), starting with 100 ng of sheared genomic DNA.

### Genome alignment

Raw sequence data in fastq format were aligned to the genome of *E. coli* strain DH10B (NCBI Reference Sequence NC_010473.1, http://www.ncbi.nlm.nih.gov/genome/167?project_id=58979) or the human reference genome hg19 (http://hgdownload.cse.ucsc.edu/downloads.html#human) using Bowtie2 [22] with the local alignment setting. For Ion Torrent sequence data, the first base of each read was trimmed to remove the ‘A’ overhang that was added during library preparation, and one mismatch was allowed during alignment (--local -- trim5 1 –N 1). For Illumina sequence data, local alignment was performed in the paired-end mode (--local). All raw sequence fastq files generated in this study have been deposited in the NCBI Sequence Read Archive (http://www.ncbi.nlm.nih.gov/sra) under project accession number SPR018873.

## Results

### Workflow for preparing NGS sequencing libraries on the AMCC chip

The design and operation of the AMCC chip is described under "Materials and methods". As a first application of the AMCC chip, we implemented on-chip protocols for preparing Next Generation Sequencing libraries from genomic DNA for the Illumina MiSeq and Ion Torrent PGM platforms. The protocols for preparing sequencing libraries from fragmented genomic DNA often include enzymatic reactions to generate blunt DNA ends, add 3'-dA tails to prevent concatemerization of genomic fragments during ligation, and ligation of dsDNA adaptors specific to the sequencing platform. Purification of the DNA is typically required after each enzymatic step.

The general workflow for preparing sequencing libraries on the AMCC chip was as follows: Fragmented genomic DNA was loaded into the sample inlet wells without prior size selection. Since the volume of the sample chamber in each reaction/purification module is only 8 nl, the sample DNA was pre-concentrated onto the bead column by flowing the sample DNA over the binding column and directing the column flow-through out to waste. The concentrated DNA on the column is then eluted into the sample chamber of the reaction circuit for the first automated reaction mix. An important aspect of the device is that for each sample that is carried through the process, the purification and reaction circuits dedicated to that sample are each reused three times. Each time the sample DNA is eluted from the column, the binding capacity of the ChargeSwitch beads or carboxylated beads is regenerated by washing the column with fresh binding buffer. In addition, the binding buffer chamber is refilled with binding buffer. During the next purification step, the reaction mix containing the sample DNA is diluted into binding buffer in the binding buffer chamber before it is loaded on the column, to reduce the sample pH to < 6.5, enabling the sample DNA to bind the ChargeSwitch beads (Figure S1 in File S1). By-products from each reaction are then removed via a waste outlet downstream of the column. For sample recovery, DNA from every reactor module is eluted into the collection wells and directly pipetted out.

### Libraries from *E. coli* genomic DNA sequencing

We assessed the efficiency of the NGS library preparation reactions performed on the AMCC chip by estimating the percentage of DNA fragments in the library with platform-specific sequencing adapters ligated on both ends. On-chip ligation reactions with Illumina adapters were performed using genomic DNA from *E. coli* strain DH10B and the resulting libraries were eluted from the chip for analysis. The absolute amount of *E. coli* genomic DNA present in the library was estimated using RT-qPCR with primer pairs designed to amplify sequences from six different regions of the *E. coli* genome (see Table S1 in File S1 for primer sequences in File S1). The absolute amount of DNA fragments with Illumina adapters ligated on both ends present in the library was estimated using RT-qPCR and a primer pair targeting the sequencing adapters. To calculate DNA concentrations based on the RT-qPCR data, standard curves were generated for each primer pair using serial dilutions of a known amount of an Illumina sequencing library prepared from *E. coli* strain DH10B. To ensure that ~100% of the DNA fragments in the library used to prepare the standard curves had Illumina adapter sequences at both ends, the library was first amplified by PCR using primers targeting the sequencing adapters. An average efficiency of approximately 75% was observed across all 14 reactors loaded with genomic DNA in a single run of the AMCC chip (Figure 2C). Reactors 8 and 16 were loaded with buffer as no template controls; no leakage of samples was observed from the neighboring reactor modules.

### Characteristics of sequencing runs on the PGM and MiSeq

To evaluate the quality of the sequencing libraries prepared on the AMCC chip, we arbitrarily selected two Ion Torrent libraries and one Illumina library for sequencing. In parallel, we sequenced the control libraries that we had prepared using the conventional benchtop protocols recommended by the manufacturers. The throughput for the PGM sequencing runs ranged from 18 to 63 megabases per library, with the variation largely attributable to the differential loading of the ion sphere particles onto the Ion 314 sequencing chip. An average of 0.31 million reads per run was obtained with read lengths ranging from 5 to 203 bp (Figure S5 in File S1). The MiSeq runs generated a higher throughput ranging from 50 to 290 megabases, based on 2 to 11 million reads with a read length of 25 bp.

**Figure 3:**
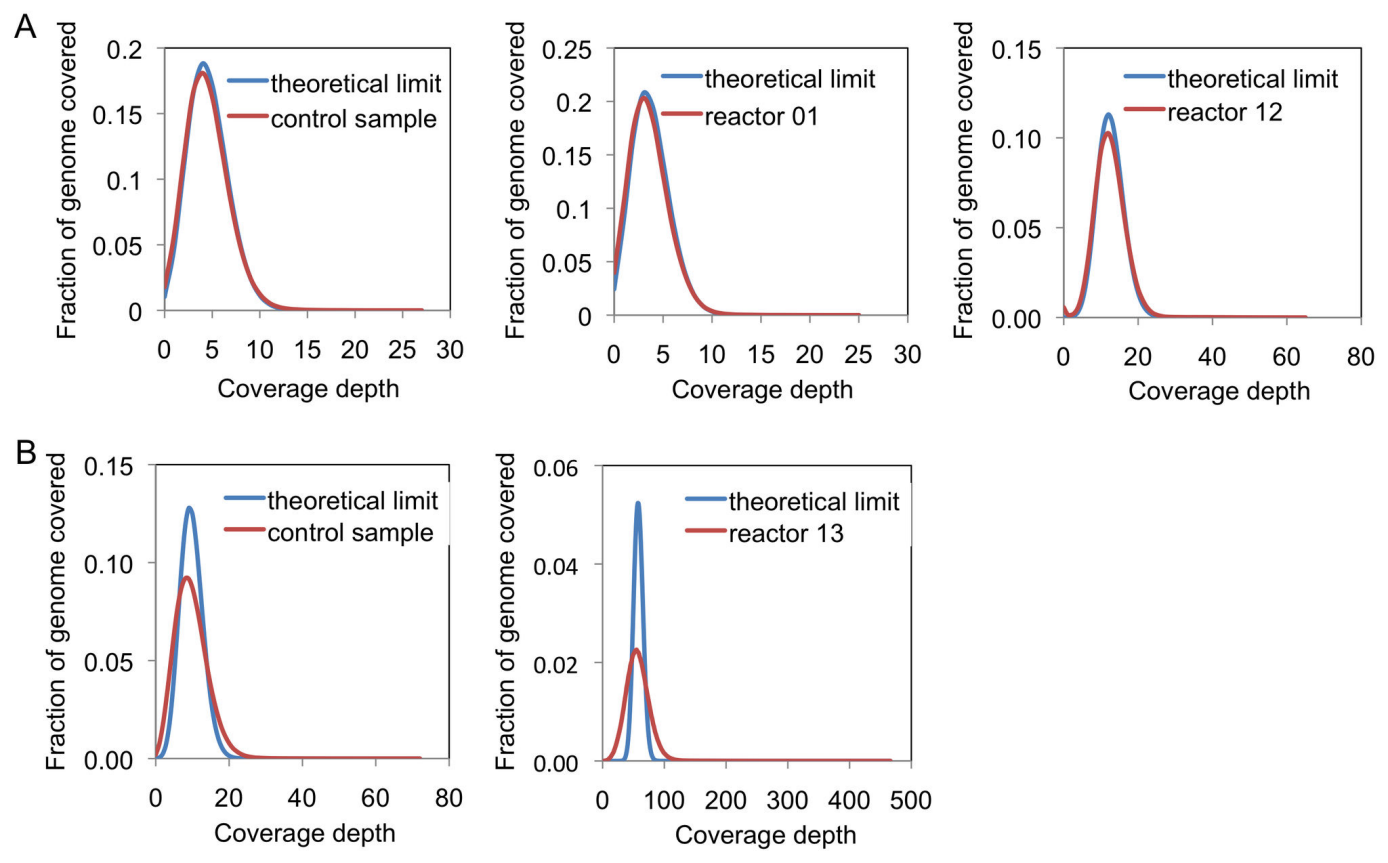
Coverage depths from sequencing runs using *E. coli* strain DH10B libraries prepared on the AMCC chip. (a) Ion Torrent libraries were run on the Ion Torrent PGM using the 100 bp sequencing protocol. (b) Illumina libraries were run on the MiSeq using the 2x25 bp paired-end sequencing protocol. Libraries labeled "Control" were prepared off-chip using the standard benchtop protocols recommended by each manufacturer. Sequencing runs with uniform coverage are expected to yield a Poisson distribution of coverage depths, indicated by the curves labeled "Theoretical limit".

The alignment rates for the reads was similar on both platforms. From the data generated on the PGM for reactors 1 and 12, there was no significant difference with the library prepared manually, each achieving an alignment rate over 97%. Sequence quality was also comparable (Figure S6A in File S1). Mean sequencing depth for reactors 1 and 12 were 3.6× and 12.5× with the control run at 4.5×. The median sequencing depths for all experimental runs as shown on Table 1 were close to the respective mean values, indicating that the distribution of the reads was not skewed in anyway. Genome coverage stood at 96% and 99% respectively for reactors 1 and 12 which were higher than the control at 92%. The distributions of coverage depth were a close fit to the theoretical Poisson distribution for all cases (Figure 3A). We estimated the library complexity for both manual and AMCC prepared libraries by randomly sub-sampling 10,000 reads from each run and determining the fraction of unique reads (Table 1). No significant differences were observed with libraries generated using either method. In addition, the sampling of reads yielded more than 99% in unique reads for all libraries. We repeated the sub-sampling analysis using sample sizes ranging from 20,000 to 100,000 and still observed similar fractions of unique reads comparing the library prepared on-chip to those prepared using the benchtop methods (Table S2 in File S1). In an independent run of the AMCC chip to prepare Ion Torrent sequencing libraries from *E. coli* strain DH10B genomic DNA, we sequenced samples derived from reactor 7 (Figure S7 in File S1). The run was successful, generating 37.18 megabases of data spanning 308 115 reads. Average sequencing depth was calculated at 7.2× after genome alignment, with 99.21% of the genome covered. Alignment rate of the reads remained high at 98.32% and the distribution of coverage depths was close to the Poisson distribution which represented the uniform coverage. These results demonstrated the efficacy of on-chip library preparation for Ion Torrent sequencing.

**Table 1:** Summary of sequencing data for the libraries prepared from *E. coli* strain DH10B genomic DNA. The *E. coli* genome is 4.69 Mbp long.

**Platform**	**Ion Torrent**				**MiSeq**	
**Protocol**	Manual*	AMCC chip run 1, reaction module 1	AMCC chip run 1, reaction module 12	AMCC chip run 2, reaction module 7	Manual*	AMCC chip run 3, reaction module 13
**Sequencing mode**	314 (100 bp)	314 (100 bp)	314 (100 bp)	314 (100 bp)	2 by 25 bp kit	2 by 25 bp kit
**Throughput (Mbp)**	20.07	18.26	62.98	37.18	50.41	290.46
**Number of reads**	210,572	151,785	566,305	308,115	2,016,250	11,618,546
**Alignment rate**	204,455 (97.10%)	147,373 (97.09%)	553,543 (97.75%)	302,950 (98.32%)	1,937,443 (96.09%)	11,485,559 (98.86%)
**Sequencing depth (mean / median)**	4.5× / 4×	3.6× / 3×	12.5× / 12×	7.2× / 7×	10.3× / 10×	61.2× / 60×
**Genome coverage**	92.23%	96.04%	99.44%	99.21%	99.80%	100%
Unique reads in a subsample**	98.4%	98.0%	98.0%	98.6%	98.6%	98.7%

* The libraries labeled "Manual" were prepared off-chip using the manufacturers’ recommended benchtop protocols.

** Subsampling for each sequencing run was performed by randomly selecting 100,000 reads for analysis. Similar results were obtained when subsampling was performed with sample sizes ranging from 10,000–100,000 reads (Table S2 in File S1).

With the Illumina libraries that we prepared on the AMCC chip, the results from on chip preparation outperformed the control run in every aspect. With a higher throughput and hence 5 fold more number of reads, sequencing depth and genome coverage were significantly higher for the on chip library prepared. Alignment rates for the reads of the control and experimental runs were 96.09% and 98.86% respectively. We generated an average 61.2× sequencing depth and achieved 100% genome coverage for this library from reactor 13. In comparison, the control run had an average 10.3× sequencing depth and 99.8% coverage. Median sequencing depth were at 60× and 10× for on chip and manual library preparation respectively, indicating the data was evenly divided around the mean. The uniformity of the runs was also confirmed with the distribution of coverage depth close to the theoretical Poisson limit as shown in Figure 3B albeit not as strongly fitted as the PGM runs. Average sequence quality per base for the MiSeq data remained high for all 25 bases of each read (Figure S6B in File S1) for both the control and on chip libraries. Uniqueness of fragments from sub-sampling 10 000 reads from each sequencing run showed a high degree of congruence with both runs having more than 98%, suggesting indifferences in library complexity using either method of library generation(Table 1). The data showed the flexibility and universality of the microfluidic device to work across different sequencing platforms.

### Library from human genomic DNA sequencing

**Figure 4:**
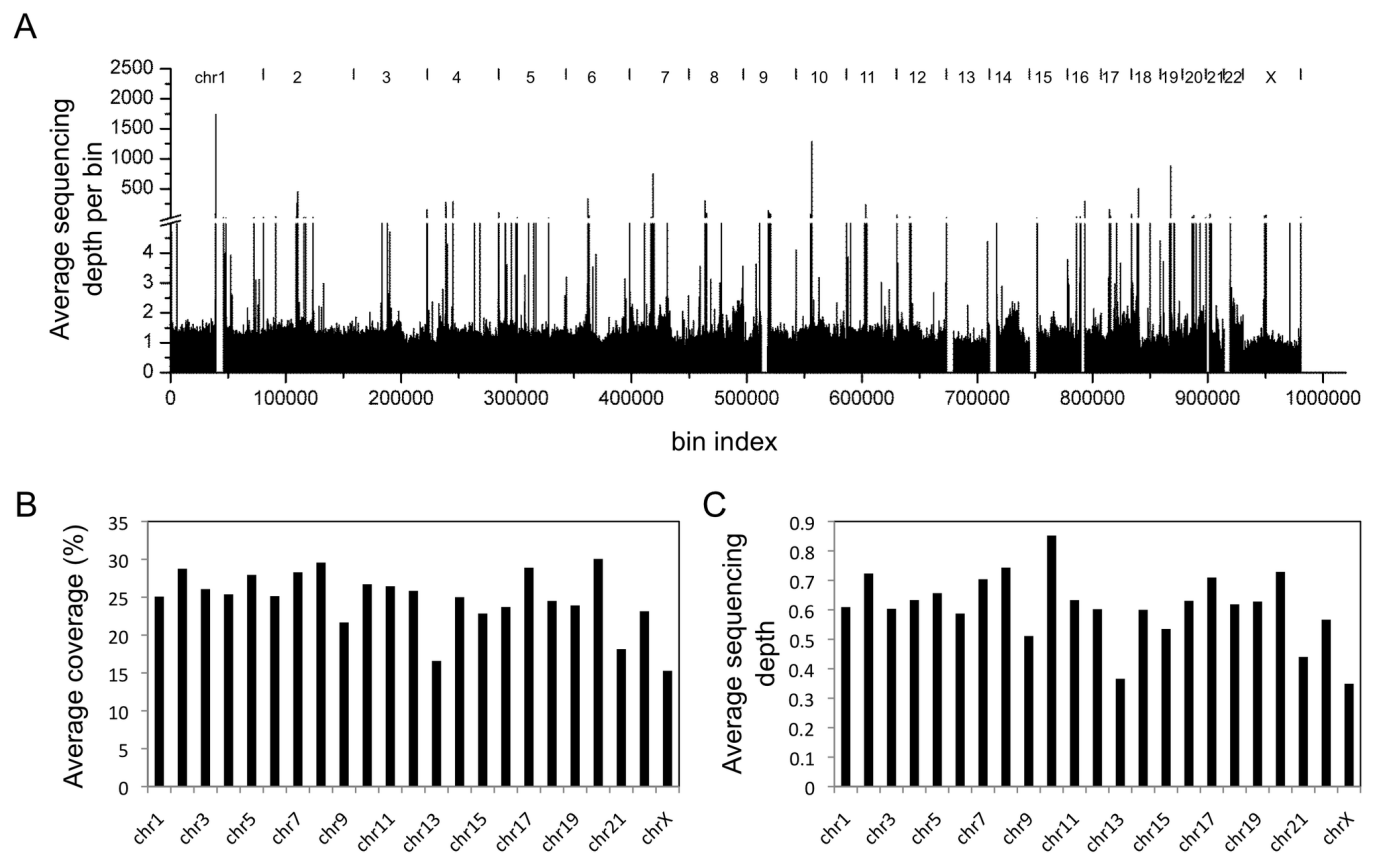
Coverage depth from a sequencing run using an Illumina U-2 OS osteosarcoma cell line library prepared on the AMCC chip. The library was run on the MiSeq using the 2x150 bp paired-end sequencing protocol. (a) Distribution of sequencing reads across the reference human genome, which has been divided into 1 million bins to assess coverage uniformity. (b) Average coverage across different chromosomes. (c) Average sequencing depth across different chromosomes. The Y chromosome is absent from the U-2 OS osteosarcoma cell line.

Using the 2 by 150 bp sequencing kit on the MiSeq, we generated approximately 10.5 million reads each for reads 1 and 2 with the elute from reactor 7. Sequencing data of approximately 3 gigabases were mapped to the reference human genome (hg19) with an alignment rate of 99.37%. To evaluate if the sequencing library provided uniform coverage of the genome, we divided the human reference genome into 1 million bins (each 3096 bp long) across all chromosomes (Figure 4A). 92.7% of the bins overlapped at least one of the sequencing reads. There was little variation in sequencing coverage and depth across different chromosomes (Figure 4B and 4C). with an average sequencing coverage of 24.7% (standard deviation 3.9% and an average sequencing depth of 0.61× (standard deviation 0.12×). These data, based on a sampling from a single sequencing run on the MiSeq, suggest that the library provided uniform coverage of the human genome and would be suitable for full-scale sequencing on a higher capacity Illumina platform such as the GAII or HiSeq.

### Reliability of column packing

To evaluate the reliability and uniformity of column packing, columns were generated with 6 cycles of a carboxylated beads mixture consisting of 15, 6, 4.5 and 3 µm beads, and we analyzed images of the resulting columns (Figure S3B in File S1). Column packing took less than 5 minutes. Column packing was relatively uniform across all 16 reaction modules in three independent runs of the AMCC chip. Bead bed volumes ranged from 48.9% to 60.5% of the column volume, with a coefficient of variation (CV) of 0.062 (Figure S3C in File S1). There was no significant difference between the mean column volumes obtained in each of the three runs (ANOVA, *p* = 0.073). These data indicate that there is excellent reproducibility of the multi-columns generation process between independent experimental runs.

### Efficiency of DNA capture

The efficiency of the columns in capturing DNA with a single pass is shown in Figures S2B and S2C in File S1 for two independent runs using three different operating conditions. The operating pressure has a strong contributory effect for DNA binding onto the columns. We observed that there is increased recovery of DNA with slower sample flow. Extended flow conditions permitted longer period of interaction for the analyte to the surface of the beads. 26 ng of template DNA is recovered when operating at 3 Psi. The maximum binding capacity of the columns was about 25 ng and the percent recovery under optimal conditions (a sample loading time of 45 min at 3 psi) ranged from 20–40% for sample inputs ranging from 1.56 ng to 100 ng, with recovery dropping to 5% for the lowest sample input of 0.78 ng. Noting that this test of DNA binding and recovery is performed with the analyte meeting the binding column only once and the design of the reactor module allows samples to be recirculated over the column multiple times (Figure S2A in File S1), the recovered DNA efficiency can be further enhanced if desired. Nonetheless, the current protocol produces sufficient DNA quantities for NGS library generation even without PCR amplification. For instance the requirements on the Illumina MiSeq is to load only 8 pM concentration of sample (or approximately 0.143 ng of the final DNA library) and the current conditions allow more than 100 fold in excess of material.

### On-chip ligation efficiency

The efficiency of the on-chip ligation reactions (defined here as the percentage of DNA fragments in the library with platform-specific sequencing adapters ligated on both ends) was estimated as follows: On-chip ligation reactions with Illumina adapters were performed using genomic DNA from *E. coli* strain DH10B and the resulting libraries were eluted from the chip for analysis. The absolute amount of *E. coli* genomic DNA present in the library was estimated using RT-qPCR with primer pairs designed to amplify sequences from six different regions of the *E. coli* genome (see Table S1 for primer sequences in File S1). The absolute amount of DNA fragments with Illumina adapters ligated on both ends present in the library was estimated using RT-qPCR and a primer set targeting the sequencing adapters (Forward primer: 5’-ACACTCTTTCCCTACACGACG-3’; Reverse primer: 5’-CTCGGCATTCCTGCTGAAC-3’). To calculate DNA concentrations based on the RT-qPCR data, standard curves were generated for each primer pair using serial dilutions of a known amount of an Illumina sequencing library prepared from *E. coli* strain DH10B. To ensure that ~100% of the DNA fragments in the library used to prepare the standard curves had Illumina adapter sequences at both ends, the library was first amplified by PCR using primers PE 1.0 and PE 2.0 (sequences provided above). The following thermal cycling conditions were used for RT-qPCR: 1 cycle of 95°C for 30 s and 35 cycles consisting of 95°C for 10 s, 60°C for 30 s and 72°C for 30 s. Fluorescence signals for two channels using SYBR Green (495 nm) and ROX (535 nm) were recovered and melting curve analysis was performed to confirm the specificity of the PCR products.

### Yield and quality of on-chip size selection

**Figure 5:**
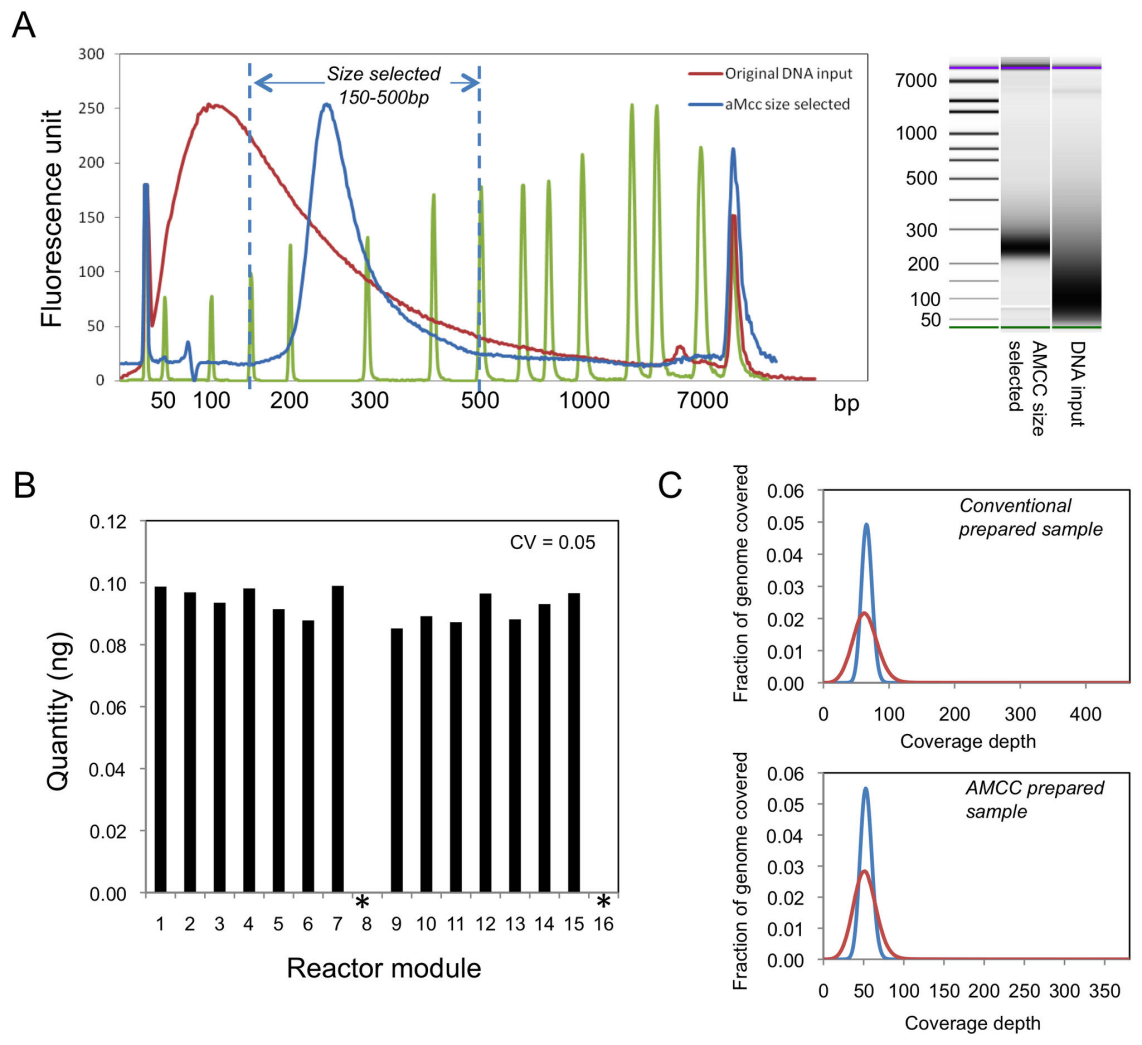
Combining NGS library preparation with size selection on the AMCC chip. Columns were formed on the AMCC chip using carboxylated beads. (A) Electropherogram showing size selection on-chip, illustrating the recovery of DNA fragments in the range of 150-500 bp. The green trace represents the DNA ladder used. GEL image extracted from a high sensitivity chip run on the Bioanalyzer 2100. (B) Quantification of Illumina library DNA eluted from the AMCC chip using RT-qPCR. Coefficient of variation (CV) for this chip run was 0.05. Asterisks indicate sample modules where buffer was loaded instead of genomic DNA. (C) Coverage depths from sequencing runs of libraries prepared on the AMCC chip and using the conventional benchtop protocol recommended by the manufacturer.

We tested the new protocol including size selection by preparing Illumina sequencing libraries using genomic DNA from *E. coli* strain DH10B. We achieved a tight selection of the desired DNA fragments suitable for MiSeq sequencing (Figure 5A). Comparing the sheared *E. coli* genomic DNA that was loaded onto the AMCC chip, which ranged from 35 to 3000 bp in length, to the library DNA eluted from the AMCC chip after on-chip size selection, which showed a narrow size range of 150-500 bp (Figure 5A).

The yields of library DNA recovered from the 14 reactors loaded with *E. coli* genomic DNA were relatively uniform across the AMCC chip (CV = 0.05), and no leakage was observed between reactors based on the absence of DNA in the recovery wells from the no template controls (reactors 8 and 16; Figure 5B). The low variability in yields contrasts somewhat to the runs of the AMCC chip using ChargeSwitch beads (Figure 2A and 2B). We attribute the greater uniformity in yields obtained with the carboxylated beads to the somewhat higher stability of the packed columns. The ChargeSwitch beads, which are much smaller than the beads in the carboxylated bead mixture, exhibited some losses during operation of the AMCC chip despite the presence of the frit layer composed of larger beads. Although the losses were not pronounced, they did increase the variability in yield between different reactors on the same chip.

To assess the quality of the libraries prepared on the AMCC chip with on-chip size selection, we sequenced one of the libraries (recovered from reactor module 13) together with a control library prepared using the standard benchtop protocol recommended by Illumina. To provide a more direct comparison of the library prepared on the AMCC chip with the control library, we performed size selection of the control library with Ampure nucleic acid purification beads (Beckman Coulter, USA), using 12.2% PEG to remove DNA fragments below the desired size range and 9.2% PEG to remove DNA fragments above the desired size range. We then sequenced both libraries on the MiSeq. Although the number of reads and sequencing throughput obtained with the AMCC library were somewhat lower than those obtained with the control library, the quality of the sequencing data was equally good based on the alignment rate, genome coverage, and percentage of unique reads, and the sequencing depth was more than adequate (Table 2). The distributions of the sequencing reads for both libraries were similar as well (Figure 5C).

**Table 2:** Summary statistics from a sequencing run of an *E. coli* strain DH10B Illumina library prepared on the AMCC chip using carboxylated beads for DNA purification and on-chip size selection.

**Library**	**Throughput (Mbp)**	**Number of reads**	**Alignment rate**	**Sequencing depth (mean / median)**	**Genome coverage**	Unique reads in a sub-sample**
Manual*	316.6	12,663,406	99.27%	69.6× / 69×	100%	99.8%
AMCC chip, reaction module 13	254.1	10,165,992	99.24%	55.8× / 55×	100%	99.8%

* The library labeled "Manual" was prepared off-chip using the standard benchtop protocol recommended by Illumina. The libraries were run on the MiSeq using the 2x25 bp paired-end sequencing protocol.

** Subsampling for each sequencing run was performed by randomly selecting 10,000 reads for analysis. Similar results were obtained when subsampling was performed with sample sizes ranging from 10,000–100,000 reads (Table S2 in File S1).

### Hands-on time

The microfluidic scale of the AMCC chip yields significant savings in the reagents used to prepare sequencing libraries compared to the standard benchtop protocols (Table 3). In addition, automation of the entire workflow using the AMCC chip greatly reduces the hands-on time required for library preparation (Table 4). The standard benchtop protocols required 120 min of hands-on time to prepare a single library. In contrast, only 20 min was required to load the AMCC chip with samples and reagents, and all subsequent steps were performed by the robotic workstation (IFC controller) unsupervised up to the point of recovering the eluted libraries. Overall, it required approximately 25 min of hands-on time to prepare up to 16 independent libraries. There was also a significant reduction in the number of pipetting steps required to prepare sequencing libraries on the AMCC chip compared to the benchtop protocols (Table 4). 

**Table 3:** Reagent usage comparing conventional benchtop and on-chip library preparation protocols.

		**Volume used per sample**	
**Protocol step**	**Enzyme mix used**	**Manual protocol**	AMCC chip protocol*
End repair	NEBNext End Repair Module	5 µL	0.31
dA Tailing	NEBNext dA-tailing module	3 µL	0.31
Adapter ligation	NEBNext Quick Ligation module	5 µL	0.31

* Reagent usage is based on loading 5 µL enzyme, which is distributed on-chip to all 16 reaction modules

**Table 4:** Library preparation workflow.

		**User hands-on time (min)**		**Incubation time (min)**		**Number of pipetting steps (for 16 samples)**	
**Protocol step**	**Manual (1 sample)**	Manual (16 samples)*	**AMCC (16 samples)**	**Manual**	**AMCC**	**Manual**	**AMCC**
Aliquoting samples/reagents		5	20			16	81
Concentrate sheared DNA					30		
End repair	10	20		30	30	4 (34)**	
DNA purification	30	30			30	80	
dA tailing	10	20		30	30	4 (34)**	
DNA purification	30	30			30	80	
Adapter ligation	10	20		15	15	5 (50)**	
DNA purification	30	30			30	80	
Sample transfer			5				16
Total	120	155	25	75	195	253 (358)	97

Time and effort spent on library preparation for a conventional benchtop protocol compared with the on-chip workflow.

* Using a multichannel pipettor and microtiter plate for assembling enzymatic reactions

** Assumes that a master mix of enzyme plus buffer is assembled and then aliquoted with a multichannel pipettor. Numbers in parentheses indicate the number of pipetting steps if a standard pipettor is used.

## Discussion

We have demonstrated that the AMCC chip can be used to perform a sequence of enzymatic reactions with intervening DNA purification steps and on-chip size selection, achieving a fully automated system to prepare NGS libraries. Central to the mechanism for library preparation on chip resided is a reusable DNA purification column that provided the flexibility to bind and release DNA with high affinity. The eluted material is then captured in the reaction circuit for subsequent enzymatic reactions. Our approach mimics that of a miniaturized purification column and the design allows for precise dispensing and straightforward construction of the columns. The use of different types of beads greatly enhances the flexibility of the microfluidic device to handle different applications. In using pH sensitive beads for DNA library preparation, we did not require the use of ethanol, chaotropic salts, organic solvents or time consuming precipitation steps. With carboxylated bead columns, we further show that DNA fragment size selection can be achieved without affecting the quality of the sequencing libraries. Importantly, we have also significantly reduced the variability in recovered DNA material with this column configuration as losses of DNA binding beads are minimized after each reaction. This enables us to produce a totally automated system for NGS sequencing library generation. Using the columns, we were able to conduct different enzymatic reactions within the same column. This eliminates the need for cascading different reactors together for the three different reactions during library preparation. Potentially, many separate reactions can be carried out in each reactor, limited only by the number of wells on the carrier dedicated to loading reagents. We have employed other space saving measures on the chip, such as designing the on-chip peristaltic pump to be universal for both circuits in each reactor, which also simplified the computer control process. The plastic carrier is designed to accommodate a device with twice as many reactors, capable of processing up to 32 samples.

The AMCC device meets the needs for high throughput preparation of DNA sequencing libraries, by allowing for the simultaneous generation of libraries from 16 different samples at the same time, with user intervention only required to load reagents and to recover the completed libraries. The microfluidic device was able to consistently and reliably produce enough sequencing material from each of the reactors yielding significant time and cost savings. We have demonstrated the cross platform compatibility of the device to prepare sequencing libraries for both the Ion Torrent PGM and the Illumina MiSeq, The quality and throughput from each of the runs were comparable to control sequencing runs that suggested the efficacy of our device at generating the libraries. In preparing samples for a more complex genome such as the human genome, the sequencing data neither showed significant sequencing bias nor non-uniformity with libraries prepared on the device. The current main device limitation for NGS library preparation is that bar-coded samples cannot be produced. Samples with low complexity or variants benefit from sample pooling for DNA sequencing to maximize the output of each run. This issue can be simply resolved by providing dedicated fluidic lines to each reactor module for different coded sequencing adapters in future design upgrades.

Other approaches have been used to automate the preparation of DNA sequencing libraries, including the use of liquid handling robots [35,37–40]. In using liquid handling robots, there is no reagent savings as reactions are generally performed using the same volumes as in the benchtop protocols. In contrast, the AMCC chip uses an order of magnitude less amount of enzyme mixes per sample which translates to effective cost savings. A microfluidic platform based on the manipulation of electrowetting droplets also exists (Mondrian SP workstation, NuGEN, USA), but this system also uses microliter volumes for reactions and therefore does not significantly reduce reagent consumption. In addition, the system does not perform size-based purification as part of its automation and requires the user to do this manually [41–43].

Various methods have been used for purifying nucleic acids within a microfluidic device, including the use of standard chaotrope chemistry on microstructures and silica-gel hybrid systems [25,26,44,45]. Low yield and large DNA elution volumes [46] were typically associated which would affect the ability to generate a concentrated sample for NGS techniques. Furthermore these purification and extraction methods required substantial preparatory processes such as complex micro-fabrication techniques to construct the microstructures and/or tedious processes to surface coat the devices. Immobilized silica particles in devices [2,47] seemed to provide better DNA recovery but had shown limited reuse capabilities to fulfill the needs of complex biochemical processes. Taking advantage of earlier work demonstrating that size selection of DNA fragments could be accomplished using carboxylated beads by varying the concentration of PEG in solution [34–36], we were able to perform size selection on the AMCC chip. In the current demonstration, we selected an intermediate range for DNA sequencing by filtering out small DNA fragments (<150 bp) and larger unwanted fragments (>500 bp. Other size ranges can be accommodated by using the right PEG concentration, offering flexibility while keeping simplicity of system operation. For applications where a tighter control of fragment sizes are desired, the AMCC system provides the ability to bypass the size selection step and the eluate can be further processed with more sensitive methods [48] such as gel size excision or pippin prep. Within the same reactor, the AMCC chip provided both the flexibility to purify and size select concurrently which is a desirable feature for NGS library preparation and associated applications such as size exclusion chromatography or sample preparation in chromatin immunoprecipitation. Micro-scale approaches for DNA size selection typically utilize capillary electrophoresis [49]. Other techniques for DNA size selection include sorting by deterministic lateral displacement of DNA [50,51] and sorting of individual DNA molecules based on fluorescence intensity of an intercalating dye [50].

The possibility of creating different compositions micro-columns allows the exploration of the AMCC chip to automate other common molecular biology techniques such as chromatin immunoprecipitation (ChIP). For instance, sepharose beads with specific antibodies can be packed into the columns on the AMCC chip to immunoprecipitate the protein-DNA complexes. DNA can then be release chemically or applying heat to the columns. Using microfluidics for this application benefits from is its higher sensitivity and the ability for low sample input [52]. With the AMCC chip, it allows full automation of the tedious tasks involved.

In conclusion, we have demonstrated the ability to perform serial nucleic acid reactions-purifications within a single reactor on a microfluidic platform. Using the device, we generated NGS sequencing libraries involving complex multiple enzymatic reactions on chip. An automated strategy is desired given the prevalence of using NGS for experimental analyses and the likelihood of requiring multiple libraries construction. With parallelization, our system produces 16 different libraries for either the Ion Torrent PGM or Illumina MiSeq at 14 times higher production rate using 9 fold reduction in reagents when compared to the conventional approach. The device has potential to be put in routine use for library preparation to eliminate the tedious and time consuming task. With the flexibility of DNA capture and release on chip and the ability to assemble complex enzymatic reactions, it should be possible to develop additional sample preparatory protocols such as chromatin immunoprecipitation (ChIP) and ChIP-seq.

## Supporting Information

File S1 **The following files are available in File S1:** **Table S1.** Primers used in this study to amplify regions of the *E. coli* genome. **Table S2.** Fraction of unique reads observed when subsampling is performed over a range of sample sizes. **Figure S1.** Reaction circuit visualized with colour dyes. **Figure S2.** DNA purification circuit visualized with colour dyes. **Figure S3.** Column generation and uniformity of columns. **Figure S4**. Quantification of *E. coli* strain DH10B library DNA after size selection and amplification by PCR. **Figure S5.** Distributions of read lengths from sequencing runs using *E. coli* strain DH10B libraries prepared on the AMCC chip. **Figure S6**. Quality scores from sequencing runs using *E. coli* strain DH10B libraries prepared on the AMCC chip. **Figure S7.** Summary statistics from a sequencing run using an *E. coli* strain DH10B Ion Torrent library prepared on the AMCC chip. **References for supporting information**.
(PDF)
