# Supplementary material for: A Microfluidic Device for Preparing Next Generation DNA Sequencing Libraries and for Automating Other Laboratory Protocols That Require One or More Column Chromatography Steps
Source: PLoS One. 2013 Jul 24;8(7):e64084. doi: 10.1371/journal.pone.0064084 (PMC3722208; doi:10.1371/journal.pone.0064084)
Supplement: File S1 — The following files are available in File S1: Table S1. Primers used in this study to amplify regions of the E. coli genome. Table S2. Fraction of unique reads observed when subsampling is performed over a range of sample sizes. Figure S1. Reaction circuit visualized with colour dyes. Figure S2. DNA purification circuit visualized with colour dyes. Figure S3. Column generation and uniformity of columns. Figure S4. Quantification of E. coli strain DH10B library DNA after size selection and amplification by PCR. Figure S5. Distributions of read lengths from sequencing runs using E. coli strain DH10B libraries prepared on the AMCC chip. Figure S6. Quality scores from sequencing runs using E. coli strain DH10B libraries prepared on the AMCC chip. Figure S7. Summary statistics from a sequencing run using an E. coli strain DH10B Ion Torrent library prepared on the AMCC chip. References for supporting information. (PDF) [file pone.0064084.s001.pdf]

Supporting material

Table S1. Primers used in this study to amplify regions of the *E. coli* genome.
Table S2. Fraction of unique reads observed when subsampling is performed over a range of sample sizes.

Figure S1. Reaction circuit visualized with colour dyes.
Figure S2. DNA purification circuit visualized with colour dyes.
Figure S3. Column generation and uniformity of columns.
Figure S4. Quantification of *E. coli* strain DH10B library DNA after size selection and amplification by PCR.
Figure S5. Distributions of read lengths from sequencing runs using *E. coli* strain DH10B libraries prepared on the AMCC chip.
Figure S6. Quality scores from sequencing runs using *E. coli* strain DH10B libraries prepared on the AMCC chip.
Figure S7. Summary statistics from a sequencing run using an *E. coli* strain DH10B Ion Torrent library prepared on the AMCC chip.

References

Table S1. Primers used in this study to amplify regions of the *E. coli* genome

Gene	Primer orientation	Sequence
mdoB	Forward	CGT TTT GCC GGT AAA GAT GT
	Reverse	GCT ACC ACG CTT TTC AGC TC
fruR	Forward	CAA CGG GGT TAT CAA CTG CT
	Reverse	GCA TCA ACC TGA CGC TGT AA
lafU	Forward	CGC TGC TGA AGA AGA AAT CC
	Reverse	AAT CCC TGG GGA ACA ATC TC
ybeM	Forward	GGA ATA TGC TGG TGG CAC TT
	Reverse	CAT CCC TTC CAC CTC CAG TA
ybfL	Forward	GGA TAC GGC ACA TCG CTA TT
	Reverse	GGC TGC TTT TCG CAT CTT AC
yhjQ	Forward	CGA TAT TTG CTC CGG CTT AC
	Reverse	CAG TTG GCA TCC ACA TTG AC

Table S2. Fraction of unique reads observed when subsampling is performed over a range of sample sizes. Data are from sequencing libraries prepared from *E. coli* strain DH10B genomic DNA.

Number of random reads selected	Fraction of unique reads in each sequencing run						
	ChargeSwitch bead columns					Carboxylated bead columns	
	PGM Manual	PGM reactor1	PGM reactor12	MiSeq Manual	MiSeq reactor13	MiSeq Manual	MiSeq reactor13
10000	0.998	0.998	0.999	0.999	0.999	0.998	0.998
20000	0.997	0.996	0.996	0.997	0.997	0.997	0.997
30000	0.995	0.994	0.994	0.996	0.996	0.995	0.996
40000	0.994	0.992	0.993	0.995	0.995	0.994	0.995
50000	0.993	0.990	0.990	0.993	0.993	0.993	0.993
60000	0.991	0.988	0.988	0.992	0.992	0.993	0.992
70000	0.988	0.986	0.986	0.991	0.990	0.991	0.990
80000	0.987	0.985	0.984	0.989	0.989	0.989	0.989
90000	0.985	0.983	0.982	0.987	0.988	0.987	0.988
100000	0.984	0.980	0.980	0.986	0.986	0.987	0.986

Supporting Figures

Figure S1. Reaction circuit visualized with colour dyes. “Sample” (yellow region) is pushed to the side of the peristaltic pump by dead end filling of the buffer before mixing. Mixing is achieved by on-chip peristaltic pumping.

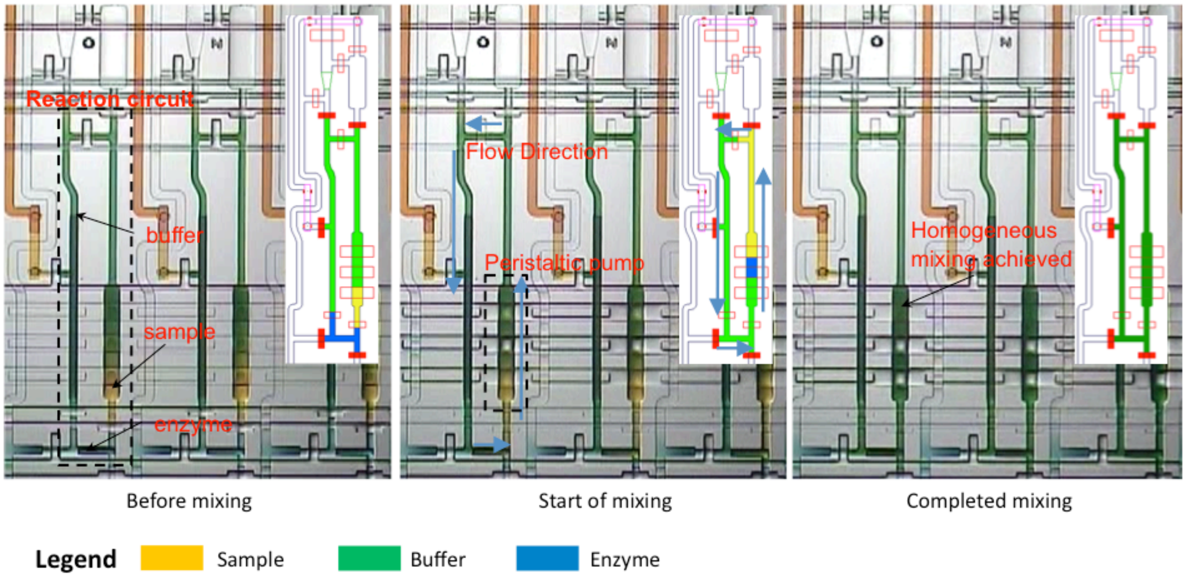

Figure S2. DNA purification circuit visualized with colour dyes. (a) DNA samples are directed over the column after mixing with binding buffer, allowing the DNA to bind to activated beads. Schematics show the change in microvalve configuration used to switch the flow of sample from the reaction circuit to the purification circuit. The reaction circuit is then ready to be air-purged for the next reaction cycle. (b) The amount of DNA bound and recovered from the columns increased as the time taken to flow the sample over the columns was increased. The loading time is determined by the pressure of the sample inlet channel, as indicated in the legend of the bar graph. 100 ng of DNA was loaded initially. (c) Percentage of DNA sample bound and recovered as a function of the amount of DNA loaded initially and the time taken to flow the samples over the columns. As in (b), the loading time is determined by the pressure applied to the sample inlet channel.

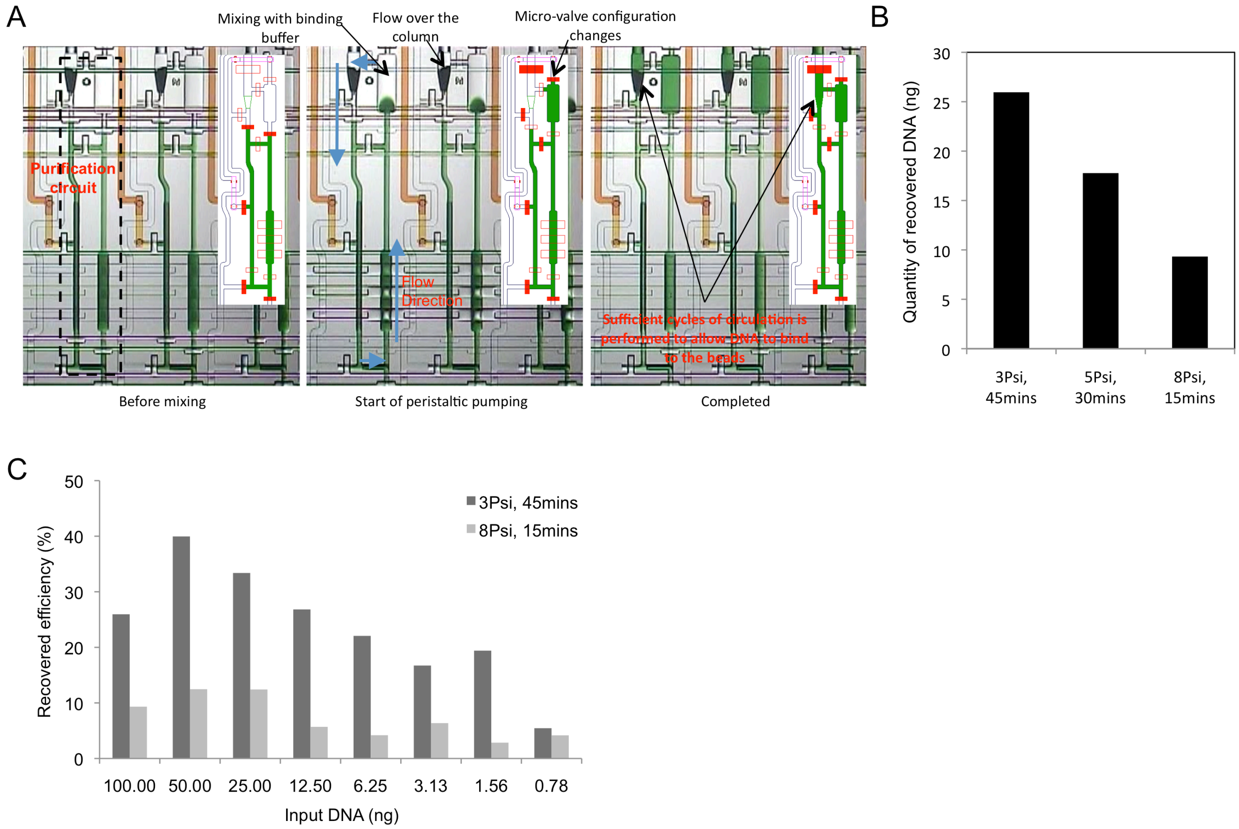

Figure S3. Column generation and uniformity of columns. (a) Formation of columns using ChargeSwitch beads. Columns are formed in a three step process consisting of a first layer of large beads followed by ChargeSwitch beads (~0.5-1 μm diameter) and a final capping layer of large beads. Columns formed are generally uniform in size. (b) Images of columns formed using carboxylated beads in each of the 16 reactor modules during a single run of the AMCC chip. (c) Uniformity of the columns formed using carboxylated beads in three independent runs of the AMCC chip. The percentage of column volume occupied by beads was estimated from the images using ImageJ [1]. The mean volume occupied by beads was 53.9%. Error bars indicate the standard error of the mean.

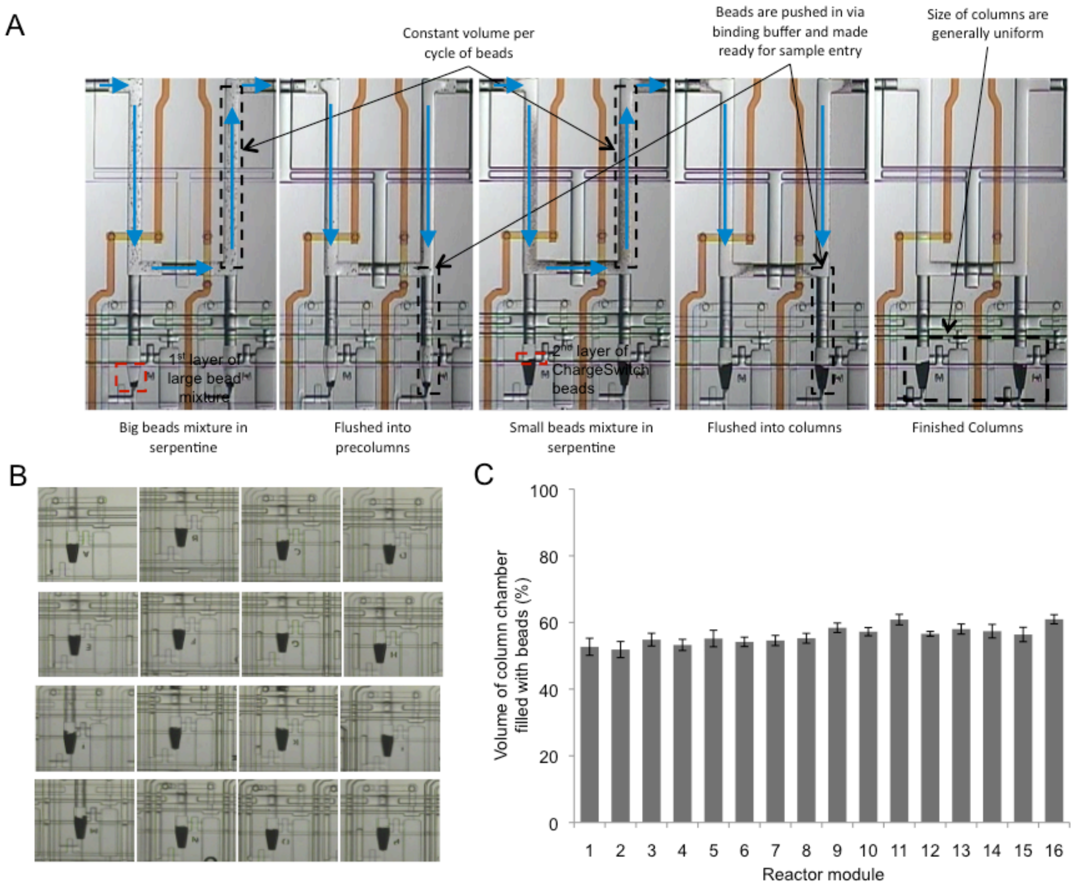

Figure S4. Quantification of *E. coli* strain DH10B library DNA after size selection and amplification by PCR: (a) Illumina libraries; (b) Ion Torrent libraries. Asterisks indicate sample modules where buffer was loaded instead of genomic DNA. Yields for the same samples after size selection but before amplification by PCR are shown in Figs. 2A and 2B.

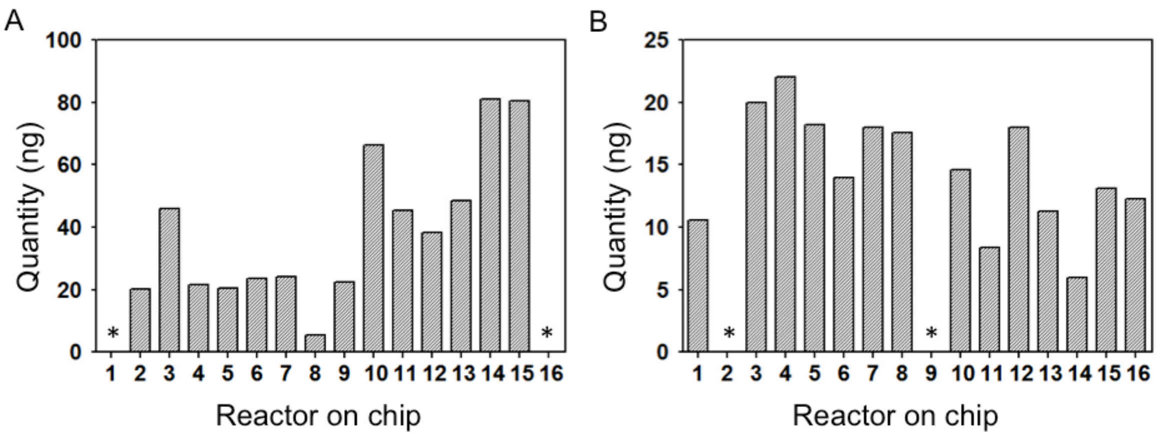

Figure S5. Distributions of read lengths from sequencing runs using *E. coli* strain DH10B libraries prepared on the AMCC chip. (a) Ion Torrent libraries were run on the Ion Torrent PGM using the 100 bp sequencing protocol. (b) Illumina libraries were run on the MiSeq using the 2x25 bp paired-end sequencing protocol. Libraries labeled "Control" were prepared off-chip using the standard bench-top protocols recommended by each manufacturer.

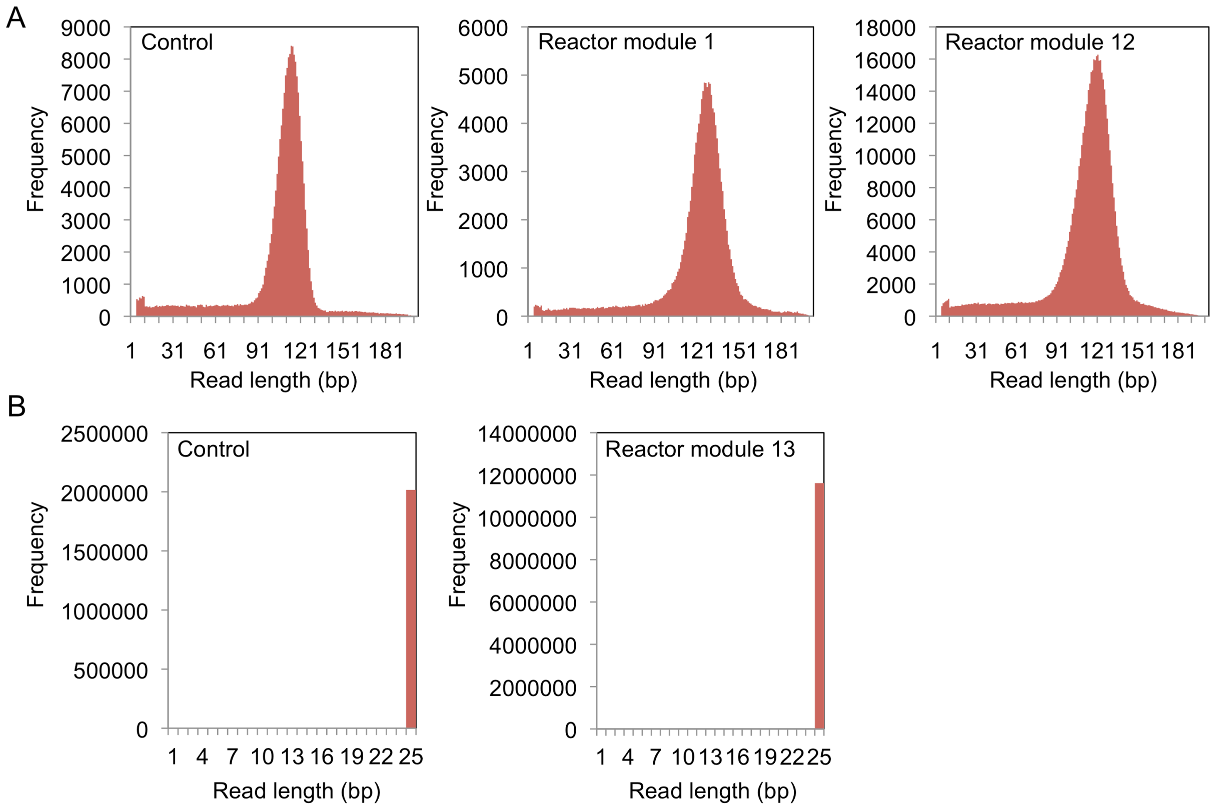

Figure S6. Quality scores from sequencing runs using *E. coli* strain DH10B libraries prepared on the AMCC chip. (a) Ion Torrent libraries were run on the Ion Torrent PGM using the 100 bp sequencing protocol. (b) Illumina libraries were run on the MiSeq using the 2x25 bp paired-end sequencing protocol. Libraries labeled "Control" were prepared off-chip using the standard bench-top protocols recommended by each manufacturer.

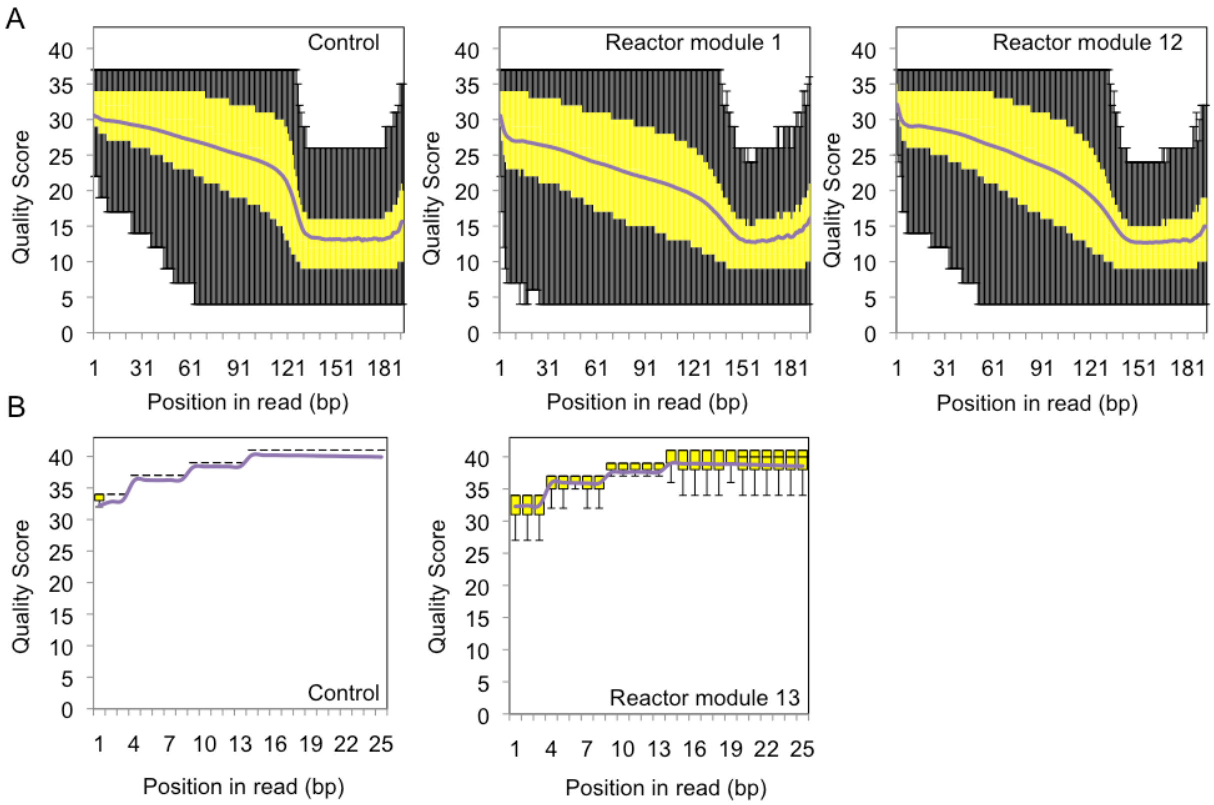

Figure S7. Summary statistics from a sequencing run using an *E. coli* strain DH10B Ion Torrent library prepared on the AMCC chip. The library was prepared in a run of the AMCC chip independent from the run used to prepare the libraries for which sequencing statistics are shown in Figs. 3, S5, and S6. The library was sequenced on the Ion Torrent PGM using the 100 bp sequencing protocol.

Description	Sequencing platform	Throughput (Mbp)	Number of reads	Alignment rate	Sequencing depth (mean / median)	Genome coverage
On chip library prep from reactor 7	314 (100bp)	37.18	308,115	(302,950) 98.32%	7.2× / 7×	99.21%

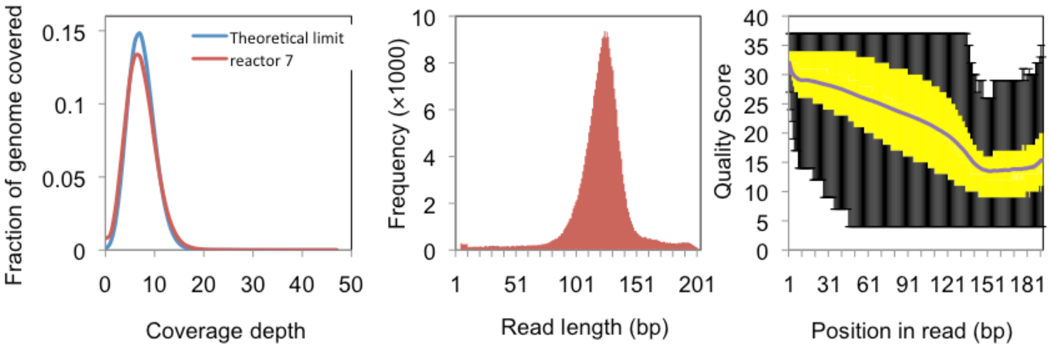

References

1. Girish V, Vijayalakshmi A (2004) Affordable image analysis using NIH Image/ImageJ. Indian J Cancer 41: 47.
